# Health digital twins as tools for precision medicine: Considerations for computation, implementation, and regulation

**DOI:** 10.1038/s41746-022-00694-7

**Published:** 2022-09-22

**Authors:** Kaushik P. Venkatesh, Marium M. Raza, Joseph C. Kvedar

**Affiliations:** Harvard Medical School, Boston, MA USA

**Keywords:** Computational models, Machine learning, Computational platforms and environments

## Abstract

Health digital twins are defined as virtual representations (“digital twin”) of patients (“physical twin”) that are generated from multimodal patient data, population data, and real-time updates on patient and environmental variables. With appropriate use, HDTs can model random perturbations on the digital twin to gain insight into the expected behavior of the physical twin—offering groundbreaking applications in precision medicine, clinical trials, and public health. Main considerations for translating HDT research into clinical practice include computational requirements, clinical implementation, as well as data governance, and product oversight.

Digital twins, or virtual models of physical objects, have been used in industry since 2002 to optimize manufacturing processes and the product life cycle^1^. However, the concept of “health digital twins” (HDT) has only recently entered health care^2^. With appropriate use, HDTs have groundbreaking applications in precision medicine, clinical trials, and public health.

Coorey et al. performed a mapping review of 88 papers related to HDT, with a particular focus on cardiovascular disease-related research. They found growing activity in HDT-related innovation, including 18 patent applications, of which 73% were from companies and 27% were from academia^2^. The authors define HDTs as a virtual representation (“digital twin”) of a patient (“physical twin”) that is generated from multimodal patient data, population data, and real-time updates on patient and environmental variables^2^. At the heart of HDT technology are two technical concepts: *cyber-physical systems* (CPS) and *closed-loop optimization* (CLO)^3^. CPS can be broken down into two components: (1) artificial intelligence (AI) systems that mimic human reasoning using big data processing and pattern recognition; (2) Internet of Things (IoT) to facilitate rapid data synchronization between physical and digital twins. CLO is the use of this real-time data to monitor, diagnose, predict disease, and optimize treatment.

The combination of AI, IoT, and CLO enables digital twins to offer predictive abilities beyond the traditional “predictor” technologies that currently exist, e.g. ICU IoT-enabled comprehensive physiological monitoring. Indeed, this HDT paradigm is a fundamental departure from traditional big data statistical techniques like logistic regressions. HDT systems generate virtual twins via *deep phenotyping*, i.e. the automated processing and integration of decentralized data compiled from patient records, biological data, and mobile sensors. The physical twin data can be used to measure and then forecast patient response to medication, behavior change, and environmental factors^2^. The real-time predictive analysis offers new opportunities for timely prevention and treatment, e.g. abdominal aortic aneurysm detection and severity classification^4^. HDTs could also be used for a cost-effective synthetic control arm for clinical trial data; Charles Fisher, CEO of Unlearn.AI, a startup that has raised over $17 million to build HDTs for trials, predicted that HDTs could reduce clinical trial expenses by 25%^5^. Aggregating HDTs to the population level, HDTs could be used for precision public health. For example, El Azzaoui et al. created HDTs of smartphone users using COVID-19 infection status and symptoms to be used for contact tracing; they also created HDTs of local hospitals for resource management and operations data sharing. They were able to combine patient and hospital HDT data to predict and optimize the flow of patients to individual hospitals in real-time^6^.

Coorey et al. identified several main challenges to translating HDT research into clinical practice, which can be conceptually categorized into three areas: computational requirements, clinical implementation, as well as data governance and product oversight^2^. The progress of AI-driven personalized medicine is contingent on *data* *fusion*, i.e. the integration of big data from several data sources containing heterogeneous information^1^. Many of these sources, such as EHR data and imaging reports, present clear obstacles to efficient data coding and sharing. HDT innovators will need to integrate data pre-processing techniques like natural language processing to overcome these integration challenges^7^. Additionally, data fusion methods should account for the varying computational complexity of real-time patient data, such as wearable data—for example, lower complexity data fusion can occur on the wearable device before exportation to the central data integration site, ultimately streamlining the process^8^. HDT technologies should ideally consider these factors when developing data management workflows.

In terms of implementation, provider adoption may be hindered by the opacity of HDT. Amid ongoing frustrations with existing technologies like EHR and common concerns of bias in AI models, transparency and education regarding HDTs are key to facilitating provider buy-in^9^. Patients should also be educated on HDTs and their purpose, potentially during the informed consent process, to bolster trust.

The adoption of these systems is also largely determined by payment considerations. HDT innovations may be financed in a variety of ways. First, HDTs may simply be a cost of business for providers if their utilization ultimately cut costs. Implementation of an HDT that improves patient outcomes could increase margins for providers participating in value-based payment schemes like episodic and bundled payments. Payers could also augment reimbursement to incentivize the use of HDTs that have been shown to achieve patient-centered and process-related outcomes^10^. Given the near-zero marginal cost of AI systems like HDTs, payers should consider looking beyond traditional fee-for-service to identify payment models that best incentivize the adoption of high-quality HDTs without resulting in overutilization which increases health care spending.

Regarding regulation, new HDT systems, or HDT “devices” as the FDA classifies them, will require a streamlined approval process. The FDA recently started the medical device development tools (MDDT) program to prequalify AI models and digital health tools, with the hopes of expediting the approval process through standardized documentation protocols^11^. HDTs offer novel complexity and data management that will require new methodologies for documentation and evaluation. Given the complexity of these technologies, regulators like the FDA should be sure to use advisory committees, including expert opinion from data scientists on the models and datasets. Regulators must also enact policies to protect patient rights, including informed consent and privacy, in this rapidly evolving innovation landscape. Given the highly detailed personal health data represented in HDT, the HDT data economy presents new threats to data privacy^12^.

Altogether, HDT technology lies at the powerful intersection of AI-powered analysis and real-time data collection, bringing health care one step closer to truly personalized medicine. Beyond individual patients, larger aggregates of patient HDTs can be used to streamline clinical trials and inform public health policy. As HDTs start penetrating clinical workflows, key considerations will include data fusion fidelity, provider and patient literacy, reimbursement, and regulation of HDTs and their secondary data economy.
